# Matrix metalloproteinase-3 promoter polymorphisms but not *dupA*-*H. pylori *correlate to duodenal ulcers in *H. pylori*-infected females

**DOI:** 10.1186/1471-2180-10-218

**Published:** 2010-08-13

**Authors:** Yi-Chun Yeh, Hsiu-Chi Cheng, Wei-Lun Chang, Hsiao-Bai Yang, Bor-Shyang Sheu

**Affiliations:** 1 Institute of Basic Medical Sciences, National Cheng Kung University Hospital, Medical College, Tainan; 2 Institute of Clinical Medicine, National Cheng Kung University Hospital, Medical College, Tainan; 3 Department of Internal Medicine, National Cheng Kung University Hospital, Medical College, Tainan; 4 Department of Pathology, National Cheng Kung University Hospital, Medical College, Tainan; 5 Department of Pathology, Ton-Yen General Hospital, Hsin-Chu, Taiwan

## Abstract

**Background:**

This study investigated if the *H. pylori dupA *genotype and certain host single nucleotide polymorphisms (SNPs) of matrix metalloproteinases (MMPs) and their inhibitors (TIMPs), including MMP-3, MMP-7, MMP-9, TIMP-1 and TIMP-2, might correlate with ulcer risk of *H. pylori-*infected Taiwanese patients.

**Results:**

Of the 549 *H. pylori-*infected patients enrolled, 470 patients (265 with gastritis, 118 with duodenal ulcer, and 87 with gastric ulcer) received SNPs analysis of MMP-3_-1612 6A > 5A_, MMP-7_-181 A > G_, MMP-9_exon 6 A > G_, TIMP-1_372 T > C _and TIMP-2_-418 G > C _by PCR-RFLP. The 181 collected *H. pylori *isolates were detected for the *dupA *genotype by PCR. The rates of *dupA*-positive *H. pylori *infection were similar among patients with duodenal ulcer (22.8%), gastric ulcer (20.0%), and gastritis (25.5%) (*p *> 0.05). Males had higher rates of duodenal ulcer and gastric ulcer than females (*p *< 0.01). Of *H. pylori*-infected patients, the MMP-3 6A6A genotype were more common in patients with duodenal ulcers than in those with gastritis (87.7% *vs*. 74.9%, *p *< 0.05) in females. This genotype had a 2.4-fold (95% CI: 1.02-5.66) increased risk of duodenal ulcer, compared to those with the 5A carrier. Combining the MMP-3/TIMP-1 genotype as 6A6A/CC, the risk of duodenal ulcer increased up to 3.6 fold (*p *< 0.05) in *H. pylori-*infected females.

**Conclusions:**

The MMP-3 promoter polymorphism, but not the *dupA*-status, may correlate with susceptibility to duodenal ulcer after *H. pylori *infection in Taiwanese females.

## Background

*Helicobacter pylori *infection leads to chronic gastritis and in some individuals, to peptic ulcer disease or even gastric carcinoma [1]. Diverse outcomes may depend on complex interactions among bacterial virulence factors, host genetics, and environmental factors [2,3]. In Taiwan, despite the nearly 100% prevalence of the so-called triple-genopositive *cagA-vacA-babA2 *virulent *H. pylori *infections, there is a lack of correlation to different disease outcomes [4,5]. It will be useful for Taiwan to validate new virulence factors or any host genomic predisposition in relation to severe *H. pylori*-infected clinical outcomes.

Recently, a duodenal ulcer-promoting gene A (*dupA*) encompassing *jhp0917 *and *jhp0918 *has been suggested to lead into higher IL-8 production of epithelial cells and thus, triggering dense neutrophil infiltration and increased risk of duodenal ulcers 2[6]. However, even in such large-scale validation, those with duodenal ulcer have a nearly 55% *dupA*-positive infection [6]. Moreover, prevalence of *dupA *and relationships between *dupA-*positive *H. pylori *and clinical outcomes are different in distinct populations [7-11]. It may indicate that *dupA *serves a promoting role leading to duodenal ulcer after *H. pylori *infection. Alternatively, it is necessary to validate host factors that predispose patients to gastroduodenal ulcer, especially with *dupA*-negative infection.

*H. pylori *infection stimulates the production of pro-inflammatory cytokines, such as IL-1, which play important roles in gastric inflammation and physiology. However, IL-1 beta or IL-1RN polymorphisms are not associated with gastric ulcer in the Taiwanese population [12].

Matrix metalloproteinases (MMPs) are a family of enzymes that degrade most extracellular matrix and correlate with ulcer formation or repairs [13]. *H. pylori *infection can up-regulate MMP-3, MMP-7, and MMP-9 in the gastric mucosa and even sera [14-16]. A large-scale German survey has further validated that the single-nucleotide polymorphisms (SNP) genotype as MMP-7_-181 _G allele and MMP-9_exon 6 _A allele increase the risk of gastric ulcer after *H. pylori *infection [17]. A deletion at MMP-3 promoter -1612, and A to G substitution at MMP-7 promoter -181 may affect transcriptional activity, leading to alterations in gene expression [18,19]. Moreover, A to G substitution at MMP-9 exon 6 causes the amino acid change required for binding to its substrate and affects its binding ability [20].

Although MMP activity is in general counteracted by endogenous tissue inhibitors (TIMPs) [21], there remains no data to check whether TIMP-1 and TIMP-2 SNP genotypes relate to the risk of gastroduodenal ulcer after *H. pylori*-infection. As such, this study surveyed if the *H. pylori dupA *genotype and certain SNP genotypes of MMP-3, MMP-7, MMP-9, TIMP-1, and TIMP-2 predispose *H. pylori*-infected Taiwanese patients to ulcer risks.

## Methods

### Patients and study design

Five hundred and forty-nine consecutive *H. pylori-*infected patients documented by upper gastrointestinal endoscopy at National Cheng Kung University Medical Center, Tainan, Taiwan were enrolled. All were genetically unrelated ethnic Han Chinese from Tainan City and the surrounding regions. None had been treated with NSAIDs, proton pump inhibitor, or any antibiotics within two weeks prior to panendoscopy on enrollment, or a past history of anti-*H. pylori *treatment and peptic ulcer. The hospital Ethics Committee approved the study.

After obtaining informed consent, 470 patients had provided enough blood samplings for SNPs analysis of MMP-3_-1612 6A > 5A_, MMP-7_-181 A > G_, MMP-9_exon 6 A > G_, TIMP-1_372 T > C _and TIMP-2_-418 G > C _by PCR-RFLP. Aside from endoscopic diagnosis for clinical diseases, at least three topographic gastric biopsies were sampled for histology or *H. pylori *culture, one each from the antrum, corpus, and cardia. These were stained with haematoxylin and eosin and reviewed for the *H. pylori*-related histology by the updated Sydney's system [4,22,23].

In addition, the study collected 181 *H. pylori *isolates for the detection of *dupA *genotype by PCR. One hundred and three isolates were collected from randomly selected patients who had agreed to undergo SNP analysis, while 78 isolates were from patients without SNP analysis. The *H. pylori *culture were conducted from the two additional gastric biopsies collected during the same endoscopy and processed with the method applied in previous publications [4,22].

For those with positive *H. pylori *culture, the isolates were extracted for genomic DNA to be analyzed for the *dupA *genotypes by PCR. The extraction of DNA was done with the same method as described previously [4,22]. Positive *H. pylori *infection was defined by positive histology or culture.

### Genotypes of SNPs in MMPs and TIMPs

Peripheral blood 8 ml was obtained from each subject for genomic DNA, which was extracted from peripheral blood mononuclear cells according to the manufacturer's instructions (Viogene, Taipei, Taiwan). Five SNPs in MMP-3_-1612 5A/6A_, MMP-7_-181 A/G_, MMP-9_exon 6 A/G_, TIMP-1_372 C/T_, and TIMP-2_-418 G/C _polymorphisms were determined by PCR-RFLP assays [18,24-26].

**Figure 1:**
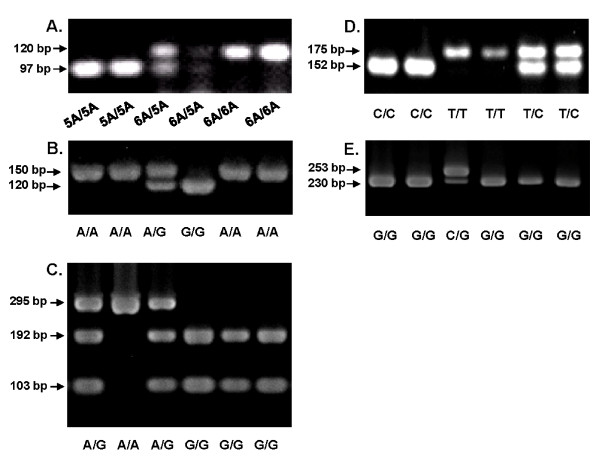
**MMP and TIMP genotyping were done by PCR-RFLP and visualized by electrophoresis on 4% agarose gel**. The listed genotype patterns were (**A**) for MMP-3 -1612 as 5A5A or 6A6A; (**B**) for MMP-7 -181 as AA, AG, or GG; (**C**) for MMP-9 exon 6 as AA, AG, or GG; (**D**) for TIMP-1 372; as CC, TC, or TT; and (**E**) for TIMP-2 -418 as CG or GG.

Using the extracted DNA as template, the regions of each MMP and TIMP were amplified by PCR using commercially available kits (GoTaq^® ^Green Master Mix, Promega, Madison, WI, USA) following the manufacturer's instructions. The sequences of primers, PCR conditions, and restriction enzymes (obtained from New England Biosciences, U.S.) used were summarized in Table 1. After digestion, the products were separated by electrophoresis on a 4% agarose gel. The MMP and TIMP genotypes were shown as different gel examples (Figure 1).

**Table 1:** The PCR primers used in the study

SNP/gene	Primer sequence (5' →3')	Size (bp)	Restriction enzyme	Reference
*MMP-3*_-1612 5A/6A_	GATTACAGACATGGGTCACG	120	*Xmn *I	Shibata *et al*, 2005
	TTTCAATCAGGACAAGACGAAGTTT		6A: 120 bp	
			5A: 97 bp + 23 bp	
*MMP-7*_-181 A/G_	TGGTACCATAATGTCCTGAAT	150	*EcoR *I	Jormsjö *et al*, 2001
	TCGTTATTGGCAGGAAGCACACAATGAATT		A: 150 bp	
			G: 120 bp + 30 bp	
*MMP-9*_exon6 A/G_	CCATCCATGGGTCAAAGAAC	295	*Sma *I	Shibata *et al*, 2005 *
	GGGCTGAACCTGGTAGACAG		A: 295 bp	
			G: 192 bp + 103 bp	
*TIMP-1 *_372 C/T_	GCACATCACTACCTGCAGTC	175	*BssSI*	Wollmer *et al*, 2002
	GAAACAAGCCCACGATTTAG		T: 175 bp	
			C: 152 bp + 23 bp	
*TIMP-2 *_-418 G/C_	CGTCTCTTGTTGGCTGGTCA	304	*BsoBI*	Zhou *et al*, 2004
	CCTTCAGCTCGACTCTGGAG		C: 253 bp + 51 bp	
			G: 230 bp + 51 bp + 23 bp	
*jhp0917*_1	TGGTTTCTACTGACAGAGCGC	307	-	Lu *et al*., 2005
*jhp0917*_2	AACACGCTGACAGGACAATCTCCC			
*jhp0917*_3	GCCTAAGACCTCAAACTAGC	296	-	This study *
*jhp0917*_4	CATTCTGTCAAGAGCTACC			
*jhp0918*_1	CCTATATCGCTAACGCGCGCTC	276	-	Lu *et al*.,
*jhp0918*_2	AAGCTGAAGCGTTTGTAACG			2005
*jhp0918*_3	GCTAGAAAGATCAACGGAAC	216	-	This study *
*jhp0918*_4	CACTTGTCTGGCTCTCAT			

*The primers were self-designed by applying Primer 3 and the restriction enzyme was selected based on the reference of Shibata *et al*, 2005.

Quality control for genotyping was achieved by including in each amplification a negative PCR control sample and three positive control samples for each SNP analyzed (homozygous for allele 1, heterozygous, and homozygous for allele 2). At least 10% of the samples were run twice in separate assays to reveal 100% concordance of the genotype designation for all of the polymorphisms. For the positive controls, the genopositive products were confirmed by direct sequencing.

### Detection of dupA gene by PCR

**Figure 2:**
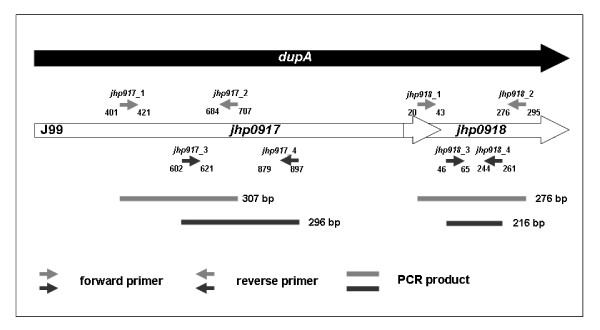
The sites of primer pairs for PCR on the *dupA *regions, including for the *jhp0917 *and the *jhp0918 *regions, applying the standard *H. pylori *isolates (J99) as reference.

The *dupA*-positive *H. pylori *was determined by positive PCR amplifications of at least 2 regions (*jhp0917 *and *jhp0918*) of the gene using two specific primer pairs (Figure 2) for strains J99 and 26695 as templates (Table 1) [6]. DNA 2 μl were added to 50-μl reaction mixture, containing 1 × PCR buffer, 1.5 mM MgCl_2_, 0.2 mM dNTP (Protech, Taiwan), 0.2 μM primers and 1 U Taq DNA polymerase (Fermentas, USA). PCR was performed with a thermal cycler (2720 thermal cycler; Applied Biosystems). The mixture was stored at 4°C and the PCR products were separated by electrophoresis on 2% agarose gel. The gels were stained with ethidium bromide and visualized under UV illumination.

### Statistical analysis

Statistical analysis was performed with the SPSS software (SPSS 12, Chicago, IL, USA). The χ^2 ^test, Fisher's exact test or Mann-Whitney U test were used as appropriate to validate the *dupA *prevalence rates, histological severity, or SNP genotypes of MMPs and TIMPs between patients with and those without ulcers. A *p *< 0.05 was taken as significant. All test significances were validated by two-tailed analysis. The odds ratios (OR) and 95% confidence intervals (CI) were used to estimate the risk to get gastroduodenal ulcer in these *H. pylori*-infected subjects.

## Results

### Demographic features of the study subjects

Of the 470 *H. pylori*-positive dyspeptic patients who provided blood samples for SNP analysis, 276 were females and 194 males, with median age of 47.2 years (range, 16-87 years). Endoscopic diagnoses included 265 with gastritis, 118 with duodenal ulcers, and 87 with gastric ulcers. Their demographics and histology after *H. pylori *infection were listed in Table 2. Patients with gastritis and duodenal ulcer had higher gastric inflammation in the antrum than those with gastric ulcers (*p *< 0.05). Among *H. pylori*-infected patients, males had higher rates of duodenal and gastric ulcers than females (51.7% *vs*. 30.9% and 58.6% *vs*. 30.9%, *p *< 0.001, respectively).

**Table 2:** Demographic and histologic characteristics of *H. pylori*-infected patients with single nucleotide polymorphism analysis (n = 470)

	Gastritis	Duodenal ulcer	Gastric ulcer	*p *value
Parameters	(n = 265)	(n = 118)	(n = 87)	
Age, mean (SD) (yr)	46.9 (13.7)	47.6 (14.0)	47.8 (11.7)	NS
Gender, n (%)				
Female: Male	183 (69.1): 82 (30.9)	57 (48.3) : 61 (51.7)	36 (41.4) : 51 (58.6)	*p *^a ^< 0.05; *p *^b ^< 0.05
Histology score, mean (SD)				
(Antrum)				
AIS (range 1-3)	1.18 (0.99)	1.39 (0.95)	0.99 (1.03)	*p *^c ^< 0.05
CIS (range 0-3)	2.34 (1.01)	2.56 (0.89)	2.05 (1.17)	*p *^a ^< 0.05; *p *^b ^< 0.05; *p *^c ^< 0.05
(Corpus)				
AIS (range 1-3)	0.85 (0.99)	0.72 (0.95)	0.86 (1.06)	NS
CIS (range 0-3)	2.24 (0.86)	2.15 (0.83)	2.13 (0.89)	NS

Abbreviations: AIS, acute inflammation; CIS, chronic inflammation. The *p *value was determined by *t *test (age), χ^2 ^test (gender) or Mann-Whitney U test (histology score). ^a ^indicated significance with *p *< 0.05 of such parameter between gastritis and duodenal ulcer; ^b ^between gastritis and gastric ulcer; ^c ^between duodenal ulcer and gastric ulcer. NS: no significant difference.

### Prevalence of dupA H. pylori infection in patients

**Figure 3:**
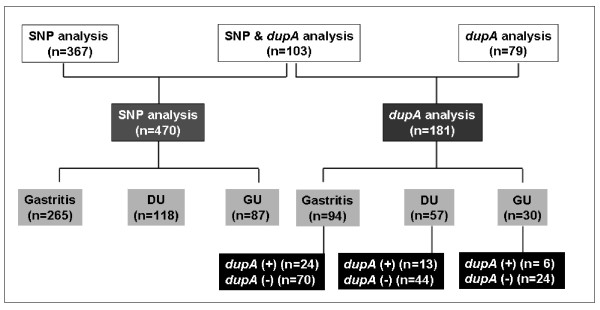
The study patients and their *H. pylori-dupA *status.

One hundred and eighty-one *H. pylori *strains were successfully obtained (Figure 3). The concordance of two PCR primer pairs was 95.3% (41/43). Only two isolates were *dupA*-positive by single primer pairs. Forty-three isolates (23.8%) were genopositive for *dupA*, of which six (20.0%) were from patients with gastric ulcer, 13 (22.8%) from patients with duodenal ulcer, and 24 (25.5%) from gastritis patients. The prevalence rates of *dupA*-positive *H. pylori *infection were similar between patients with and those without ulcers (*p *> 0.05).

### MMP and TIMP genotypes and the H. pylori-related gastro-duodenal ulcer

The 470 *H. pylori*-infected patients with SNP analysis had > 99% average genotyping success and the distributions of all SNPs were in Hardy-Weinberg equilibrium (*p *> 0.05). Since the ulcer rate had gender differences (Table 2), five genotype distributions were analyzed and separated by gender. There were no significant differences in genotype distributions of MMP-7_-181 A/G_, MMP-9_exon 6 A/G_, TIMP-1_372 T/C _and TIMP-2_-418 G/C _between patients with different clinical diagnoses (*p *> 0.05) (Table 3).

**Table 3:** The SNP genotypes of MMPs and TIMPs in the both genders with different clinical diagnoses

Genotype		Female					Male				
			
N (%)		Gastritis	DU	GU	***P ***^***a***^	***P ***^***b***^	Gastritis	DU	GU	***P ***^***a***^	***P ***^***b***^
MMP-3											
5A carrier		46 (25.1)	7 (12.3)	8 (22.2)	**0.04**	0.71	28 (34.1)	15 (24.6)	11 (21.6)	0.22	0.12
6A/6^a^		137 (74.9)	50 (87.7)	28 (77.8)			54 (65.9)	46 (75.4)	40 (78.4)		
MMP-7											
A/A		150 (82.9)	45 (80.4)	32 (91.4)		0.31	72 (88.9)	52 (86.7)	39 (76.5)	0.69	0.06
G carrier		31 (17.1)	11 (19.6)	3 (8.6)	0.67		9 (11.1)	8 (13.3)	12 (23.5)		
MMP-9											
A carrier		78 (42.6)	19 (33.3)	15 (41.7)	0.21	0.92	25 (30.5)	22 (36.1)	22 (43.1)	0.48	0.14
G/G		105 (57.4)	38 (66.7)	21 (58.3)			57 (69.5)	39 (63.9)	29 (56.9)		
TIMP-1											
♀ T carrier	♂ T	148 (80.9)	41 (73.2)	29 (80.6)	0.22	0.97	46 (56.8)	31 (50.8)	26 (51.0)	0.48	0.51
C/C	C	35 (19.1)	15 (26.8)	7 (19.4)			35 (43.2)	30 (49.2)	25 (49.0)		
TIMP-2											
C carrier		54 (30.2)	17 (32.1)	10 (28.6)	0.79	0.85	27 (33.3)	20 (32.3)	13 (25.5)	0.89	0.34
G/G		125 (69.8)	36 (67.9)	25 (71.4)			54 (66.7)	42 (67.7)	38 (74.5)		

Abbreviations: DU, duodenal ulcer; GU, gastric ulcer. The *p *value was determined by Fisher's exact test or χ^2 ^test. ^a ^indicated significance with *p *< 0.05 of such parameter between gastritis and duodenal ulcer; ^b ^between gastritis and gastric ulcer. Genotype distribution of SNP in cases and control was in Hardy-Weinberg equilibrium (*p *< 0.05).

There was a higher rate of MMP-3 6A6A genotype in patients with duodenal ulcers than in patients with gastritis (87.7% *vs*. 74.9%, *p *< 0.05). *H. pylori*-infected subjects with the MMP-3 6A6A genotype had a 2.4-fold (95% CI: 1.02-5.66) increased risk of duodenal ulcer in females compared to those with the 5A carrier. Because TIMP-1 genotypes modulated MMP-3 activity, it was further tested whether the MMP-3_-1612_/TIMP-1_372 _Combined genotypes contributed to increased risk of duodenal ulcers in females. The combined MMP-3/TIMP-1 genotype as 6A6A/CC had a 3.6-fold (*p *< 0.05) increased risk of duodenal ulcer in *H. pylori-*infected female (Table 4).

**Table 4:** Risks of combined MMP-3/TIMP-1 genotype for developing duodenal ulcer in females

	Gastritis	Duodenal ulcer	OR (95% CI)	*P*
**MMP-3**_**-1612**_**- TIMP-1**_**372**_	n (%)	n (%)		
5A carrier - T carrier	39 (88.6)	5 (11.4)	1	-
5A carrier - C/C	7 (77.8)	2 (22.2)	2.23 (0.36 - 13.85)	0.59
6A/6A - T carrier	109 (75.2)	36 (24.8)	2.58 (0.94 - 7.03)	0.06
6A/6A - C/C	28 (68.3)	13 (31.7)	3.62 (1.16 - 11.32)	0.03

The *p *value was determined by Fisher's exact test. OR, odds ratio; 95% CI, 95% confidence interval.

## Discussion

This study surveyed whether the bacterial factor *dupA *in *H. pylori *or single nucleotide polymorphisms of MMPs and TIMPs correlated with the susceptibility of gastroduodenal ulcers after *H. pylori *infection. It shows a rather low prevalence (23.8%) of *dupA*-positive *H. pylori *infection in Taiwan. Moreover, such a low prevalence limits its association to susceptibility to gastroduodenal ulcers after *H. pylori *infection.

The negative finding is consistent with several studies worldwide [8,10,11] but differs from Lu et al., who support the promoting role of *H. pylori dupA *[6]. In their positive report [6], *dupA*-positive prevalence is nearly 50% and it can be attributed as the promoting role of ulceration. Their data suggest that there should be some other bacterial virulence factor of *H. pylori *as *CagA, babA2, vacA*, or host factors, which determine the susceptibility of ulceration. In Taiwan, there is nearly a 100% prevalence of *CagA, babA2, vacA *triple-positive infection [4,15]. The current study area should be a good place to validate the host factor predisposing to ulcer risk.

In the *in vitro *promoter functional assay of fibroblasts and vascular smooth muscle cells, the MMP-3 -1612 as 5A allele has greater promoter activities than the 6A allele [19]. This implies that patients carrying the lower promoter activity genotype 6A6A in the MMP-3 promoter are accompanied by lower MMP-3 expressions of the gastric mucosa. This study discloses the host genotype MMP-3 -1612 as 6A6A, which expresses lower MMP-3 carries a 2.4-fold risk of having duodenal ulcers among females after *H. pylori *infection (*p *< 0.05) (Table 3). Moreover, TIMP-1 372, as CC, contributes a higher risk of duodenal ulcers to MMP-3 -1612 6A6A (Table 4). This data suggests that patients with higher MMP-3 expression may have lower ulcer risk, but the reasons remain uncertain.

In general, MMP-3 can degrade a wide range of substrates, including fibronectin, type IV, V, IX, and X collagens, elastin, laminins, gelatin, and proteoglycan core proteins, and is thus helpful during wound healing of the skin [27-29]. Moreover, the gastric mucosa at the ulcer site also has significantly higher expression of MMP-3 than those in the antrum [30], which suggests MMP-3 is abundant in the ulcer part, and this may help the process of re-epithelialization and contribute to wound healing.

Hellmig et al. disclose the positive associations of MMP-7 promoter -181 and MMP-9 exon 6 SNPs to the presence of gastric ulcer among Germans [17]. However, this may be due to distinct ethnic or racial variations and such positive linkage is not disclosed in the current study from Taiwan.

This is the first report to show that there is no direct association between the genotypes of TIMP-1 372 at exon 5 and TIMP-2 at promoter -418, and the presence of gastroduodenal ulcers (Table 3). However, because TIMP-1 genotypes may modulate MMP-3 activity, further testing if the MMP-3_-1612 _/TIMP-1_372 _Combined genotypes contribute to increased risk of duodenal ulcers shows that the combined MMP-3/TIMP-1 genotype as 6A6A/CC has a 3.6-fold increased risk of duodenal ulcer (*p *< 0.05) in *H. pylori-*infected females. This data suggests that TIMP-1 may also have a supportive role in interacting with MMP-3 during ulcerogenesis by *H. pylori *infection, especially in females.

However, the exact reason why such a combined MMP-3/TIMP-1 genotype as 6A6A/CC has just an increased risk of duodenal ulcer in *H. pylori-*infected females, but not in male, remains uncertain. If such a regulation between MMP-3 and TIMP-1 play a role in female's ulceration, our data at least imply some other host or environment factors in male should be more dominant than such combined genotype.

The study has a limitation of just providing 181 isolates for the analysis of the *dupA *status of *H. pylori*, which disclose a rather low 20% *dupA*-positive prevalence rate. Accordingly, the study became limited to only 103 patients to provide both analyses on the infected isolate's *dupA *status and the host's SNPs (Figure 2). It thus cannot provide an adequate statistical power to determine the exact impact of MMP-3 SNPs under *dupA*-negative specific conditions.

## Conclusions

In conclusion, this study provides evidence that host promoter polymorphisms of MMP-3 contribute to increased individual susceptibility to duodenal ulcers in females after *H. pylori *infection in Taiwan. The MMP-3 promoter genotypes may serve to screen out patients at risk and target for *H. pylori *eradication in order to stop the ulceration process among *H. pylori*-infected patients without ulcers yet.

## Authors' contributions

YCY prepared the manuscript, and carried out the molecular genetic studies to the host SNPs and *dupA *genotyping for the collected isolates of *H. pylori*. HCC and WLC carried out the SNP analysis and clinical specimen collection during endoscopy. HBY participated in the design of the study, performed the analysis of pathology, and statistical analysis. BSS conceived of the whole study, and participated in its design and coordination. All authors read and approved the final manuscript.
